# Prognostic value of routine laboratory variables in prediction of breast cancer recurrence

**DOI:** 10.1038/s41598-017-08240-2

**Published:** 2017-08-15

**Authors:** Zhu Zhu, Ling Li, Zhong Ye, Tong Fu, Ye Du, Aiping Shi, Di Wu, Ke Li, Yifan Zhu, Chun Wang, Zhimin Fan

**Affiliations:** 1 Department of Breast Surgery, The First Hospital of Jilin University, Changchun, Jilin 130021 China; 2 Department of Medical Oncology, Thomas Jefferson University, Philadelphia, PA 19107 USA; 3 Department of Emergency, The First Hospital of Jilin University, Changchun, Jilin 130021 China; 4 Department of Environmental Health, School of Public Health, Nantong University, Nantong, Jiangsu 226000 China

## Abstract

The prognostic value of routine laboratory variables in breast cancer has been largely overlooked. Based on laboratory tests commonly performed in clinical practice, we aimed to develop a new model to predict disease free survival (DFS) after surgical removal of primary breast cancer. In a cohort of 1,596 breast cancer patients, we analyzed the associations of 33 laboratory variables with patient DFS. Based on 3 significant laboratory variables (hemoglobin, alkaline phosphatase, and international normalized ratio), together with important demographic and clinical variables, we developed a prognostic model, achieving the area under the curve of 0.79. We categorized patients into 3 risk groups according to the prognostic index developed from the final model. Compared with the patients in the low-risk group, those in the medium- and high-risk group had a significantly increased risk of recurrence with a hazard ratio (HR) of 1.75 (95% confidence interval [CI] 1.30–2.38) and 4.66 (95% CI 3.54–6.14), respectively. The results from the training set were validated in the testing set. Overall, our prognostic model incorporating readily available routine laboratory tests is powerful in identifying breast cancer patients who are at high risk of recurrence. Further study is warranted to validate its clinical application.

## Introduction

Breast cancer is currently the most frequently diagnosed cancer and the leading cause of cancer-related mortality in women. Excluding skin cancers, breast cancer accounts for nearly 1 in 3 cancers^1^. In 2015, an estimated 231,840 new cases of invasive breast cancer will be diagnosed among women in the U.S., and approximately 40,290 women are expected to die from breast cancer^2^. Overall breast cancer death rates decreased 36% from 1989 to 2012 due to improvements in early detection and systemic therapies^2–4^. However, recurrence is still a major concern after surgical removal of primary breast tumor. Most locoregional failures occur within 5 years^5^. Both ipsilateral breast tumor recurrence and other locoregional recurrences are associated with significantly increased risk of distant disease and death^5, 6^.

A number of clinical and biological prognostic factors, such as age, performance status, sites of disease, hormone receptor status, and therapies, are associated with long-term clinical outcomes among women with breast cancer^7^. At present, the prognosis, classification, and treatment of breast cancer are dependent on tumor histological grade, lymph node stage, tumor stage, as well as 3 major protein markers: estrogen receptor (ER), progesterone receptor (PR), and human epidermal growth factor receptor 2 (HER2)^4, 8^. Several recent studies incorporated various genetic and molecular biomarkers to develop new prognostic models for breast cancer^9^. Nevertheless, most of the markers are not yet available in routine clinical practice, and their applicability may be limited by high cost and the need for specialized equipment and expertise. Therefore, development of novel prognostic models based on easily available markers from routine clinical practice, will benefit oncologists in identifying patients at risk of locoregional recurrences and distant metastases so as to utilize more efficient patient-tailored treatment strategies.

In this study we hypothesized that a model incorporating biomarkers from conventional laboratory tests may provide valuable information on breast cancer prognosis. To test this hypothesis, we analyzed the associations between 33 routine blood-based laboratory tests and disease free survival (DFS) of patients with breast cancer. Incorporating variables which were significantly associated with DFS in univariate analysis into our prognostic model could better stratify patients into different risk groups. Thus, we offer a new prognostic model which is a noninvasive and inexpensive tool to aid physicians in estimating patient survival.

## Results

### Characteristics of study population

**Figure 1:**
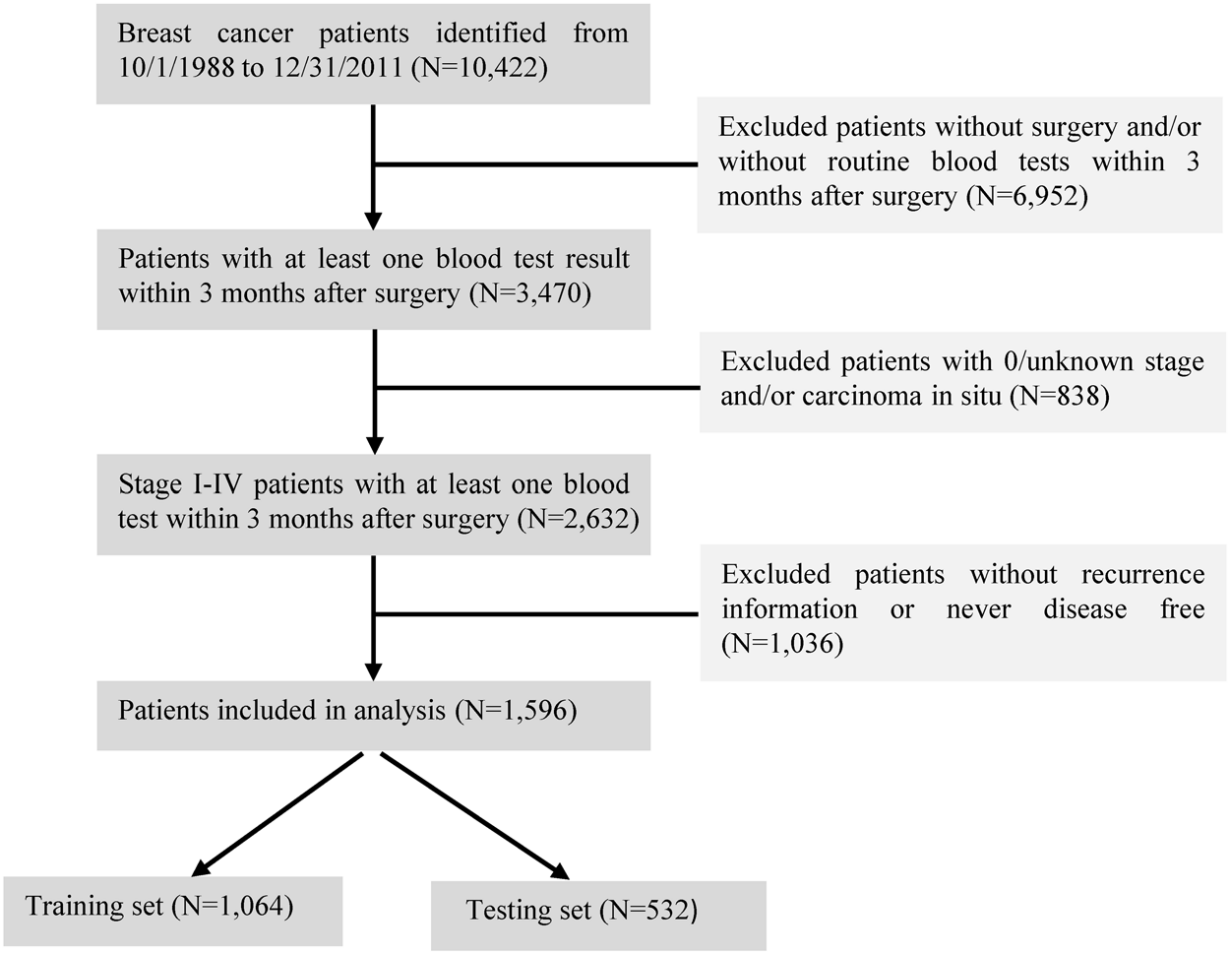
Diagram of Study Population Selection. All of patients were histologically confirmed as having breast cancer and were diagnosed and/or treated in the Kimmel Cancer Center at the Thomas Jefferson University Hospital. A cohort of 1,596 breast cancer patients was included in this study based on the selection criteria.

A total of 1,596 histologically confirmed breast cancer patients were included in this study. The detailed selection criteria are depicted in Fig. 1. Among the 1,596 patients, 1,053 (66.0%) patients were recurrence-free during follow-up, and 543 (34.0%) patients had recurrent disease or died. The patients were divided into a training set (N = 1,064) and a testing set (N = 532). The median follow-up time was 3.6 years (interquartile range [IQR] 1.8–8.2) and 4.2 years (IQR 2.0–8.3) in the training and testing set, respectively (*P* = 0.30). Demographic and basic clinical variables are summarized in Table 1. The differences between the training and testing sets were not statistically significant for almost all the demographic and clinical variables, except for tumor grade with a borderline significance (*P* = 0.04). The mean values of 33 laboratory variables are listed in Supplementary Table S1. A total of 12 variables with more than 50% missing observations were excluded from further analysis. The missing values of the remaining 21 variables ranged from 3.0% to 45.8% in the training set.

**Table 1:** Characteristics of patients in the training and testing sets.

Patients characteristic	Training set N = 1,064 (%)	Testing set N = 532 (%)	*P* value
Age, mean ± SD, (years)	59.0 ± 14.0	59.6 ± 12.9	0.35
Race/ethnicity			0.91
Caucasian	777 (73.0)	390 (73.3)	
African American	214 (20.1)	112 (21.0)	
Asian	42 (4.0)	17 (3.2)	
Others	12 (1.1)	5 (0.9)	
Unknown	19 (1.8)	8 (1.5)	
Smoking status			0.55
Never smoking	571 (53.7)	265 (49.8)	
Current smoking	124 (11.6)	67 (12.6)	
Former smoking	215 (20.2)	116 (21.8)	
Unknown	154 (14.5)	84 (15.8)	
Drinking status			0.99
Never drinking	482 (45.3)	237 (44.5)	
Current drinking	380 (35.7)	192 (36.1)	
Former drinking	6 (0.6)	3 (0.6)	
Unknown	196 (18.4)	100 (18.8)	
Tumor stage			0.73
Stage I	566 (53.2)	275 (51.7)	
Stage II	355 (33.4)	190 (35.7)	
Stage III	112 (10.5)	53 (10.0)	
Stage IV	31 (2.9)	14 (2.6)	
Tumor grade			0.04
Well differentiated	121 (11.4)	87 (16.4)	
Moderately differentiated	410 (38.5)	201 (37.8)	
Poorly differentiated	395 (37.1)	178 (33.5)	
Not determined	138 (13.0)	66 (12.4)	
Tumor histology			0.80
Invasive ductal carcinoma	868 (81.6)	438 (82.3)	
Invasive lobular carcinoma	74 (6.9)	38 (7.1)	
Mixed carcinoma	106 (10.0)	46 (8.7)	
Others^a^	16 (1.5)	10 (1.9)	
Tumor size			0.47
2–9 mm	181 (17.0)	98 (18.4)	
10–29 mm	447 (42.0)	232 (43.6)	
30–49 mm	106 (10.0)	54 (10.1)	
50–99 mm	71 (6.7)	28 (5.3)	
≥10 cm	13 (1.2)	2 (0.4)	
Unknown/not found	246 (23.1)	118 (22.2)	
Lymph nodes metastatic rate			0.50
0%	591 (55.5)	289 (54.3)	
1–20%	128 (12.0)	77 (14.5)	
20–49%	55 (5.2)	31 (5.8)	
50–79%	32 (3.0)	23 (4.3)	
80–100%	34 (3.2)	15 (2.8)	
Not determined	176 (16.5)	76 (14.3)	
Unknown	48 (4.5)	21 (4.0)	
Estrogen receptor status			0.77
Negative	223 (21.0)	104 (19.6)	
Positive	734 (69.0)	371 (69.7)	
Unknown	107 (10.0)	57 (10.7)	
Progesterone receptor status			0.76
Negative	313 (29.5)	154 (28.9)	
Positive	642 (60.3)	317 (59.6)	
Unknown	109 (10.2)	61 (11.5)	
Chemotherapy			0.97
No	618 (58.1)	307 (57.7)	
Yes	414 (38.9)	208 (39.1)	
Unknown	32 (3.0)	17 (3.2)	
Radiation therapy			0.12
No	603 (56.7)	290 (54.5)	
Yes	427 (40.1)	214 (40.2)	
Unknown	34 (3.2)	28 (5.3)	
Hormone therapy			0.64
No	652 (61.3)	323 (60.7)	
Yes	339 (31.9)	178 (33.5)	
Unknown	73 (6.9)	31 (5.8)	

Abbreviations: SD, standard deviation.

^a^Others include colloid, medullary, tubular, papillary carcinoma, and Paget’s disease.

### Univariate analysis

Kaplan-Meier and univariate Cox proportional hazards regression analysis were used to select candidate variables to be included in stepwise selection. Ten demographic and basic clinical variables (age, race, tumor stage, tumor size, lymph nodes metastatic rate, ER status, PR status, chemotherapy, radiation therapy, and hormone therapy) were significantly associated with DFS (Supplementary Table S2). Among the remaining 21 laboratory variables, 8 exhibited significant associations with DFS in a univariate basis (Table 2), including HCT, HGB, RBC, and RDW from the CBC panel, albumin and ALP from the CMP panel, and INR and PT from the coagulation panel. All of these 8 variables were significant when they were analyzed as both categorical and continuous variables, as well as in log-rank analysis. They were included as candidate prognostic factors in the next step of stepwise selection and model construction.

**Table 2:** Candidate laboratory variables selected by univariate analysis in the training set.

Variables^a^	% of missing value	No. of patients disease free/recurrence	HR (95% CI)	Cox *P*	Log-rank *P*	Brootstrap % (<0.05)
HCT	3.57				<0.0001	98.1
≤37.48%		295/218	1.00			
>37.48%		358/155	0.65 (0.53–0.80)	<0.0001		
HGB	3.48				<0.0001	99.7
≤12.50 T/L		306/211	1.00			
>12.50 T/L		348/162	0.62 (0.51–0.76)	<0.0001		
RBC	3.01				<0.0001	97.9
≤4.19 T/L		300/216	1.00			
>4.19 T/L		358/158	0.67 (0.54–0.82)	0.0001		
RDW	3.57				<0.0001	100.0
≤13.40%		371/160	1.00			
>13.40%		282/213	1.85 (1.51–2.27)	<0.0001		
Albumin	36.09				0.03	70.1
≤4.30 g/dL		266/115	1.00			
>4.30 g/dL		191/108	0.74 (0.57–0.97)	0.03		
ALP	30.64				0.002	87.4
≤69 IU/L		268/111	1.00			
>69 IU/L		224/135	1.48 (1.15–1.91)	0.002		
INR	39.85				<0.0001	99.9
≤1.02		234/99	1.00			
>1.02		175/132	2.00 (1.54–2.60)	<0.0001		
PT	45.77				<0.0001	100.0
≤13.55		214/75	1.00			
>13.55		147/141	2.14 (1.61–2.85)	<0.0001		

Abbreviations: RBC, red blood cell; HGB, hemoglobin; HCT, hematocrit; RDW, red cell distribution width; INR, international normalized ratio; ALP, alkaline phosphatase; HR, hazard ratio; CI, confidence interval.

^a^Variables were categorized by the median value in the study population.

### Stepwise selection and final model construction

Multiple imputation method was used to generate 10 imputed datasets from the training set, and stepwise selection was conducted forward to identify the best group of variables to be included in the multivariate Cox proportional hazards model for each imputed dataset. The number of times that each of the 8 variables was selected for inclusion in the model by stepwise selection is summarized in Supplementary Table S3. Three variables (HGB, ALP, and INR) which were selected from ≥6 imputed datasets were included in the final model. The parameter estimates (regression coefficients or weights) and standard errors of the 10 significant demographic and basic clinical variables (age, race, stage, tumor size, lymph nodes metastatic rate, ER status, PR status, chemotherapy, radiation therapy, and hormone therapy), as well as, 3 laboratory variables (HGB, ALP, and INR) in the final model are showed in Table 3. As showed in Supplementary Table S4, the prognostic index was calculated for each patient based on the final model.

**Table 3:** Parameter estimates and standard errors in the final model.

Variables	Coefficients	SE
Age	0.03	0.004
Race (Caucasian as reference)		
African American	0.21	0.13
others	0.15	0.23
Stage (stage I as reference)		
II	0.29	0.17
III	1.07	0.23
IV	1.87	0.28
Tumor size (2–9 mm as reference)		
10–29 mm	−0.07	0.20
30–49 mm	−0.11	0.15
50–99 mm	0.06	0.21
≥10 cm	0.08	0.21
Unknown/not found	0.07	0.40
Lymph nodes metastatic rate (0% as reference)		
1–20%	−0.11	0.20
20–49%	−0.15	0.26
50–79%	−0.15	0.31
80–100%	0.38	0.26
Not determined	0.08	0.17
Unknown	0.06	0.23
ER status (Negative as reference)		
Positive	−0.18	0.20
Unknown	0.14	1.05
PR status (Negative as reference)		
Positive	−0.15	0.18
Unknown	−0.13	1.04
Chemotherapy (without chemotherapy as reference)		
With chemotherapy	0.08	0.42
Unknown	0.02	0.14
Radiation therapy (without radiation therapy as reference)		
With radiation	0.26	0.35
Unknown	−0.43	0.12
Hormone therapy (without hormone therapy as reference)		
With hormone	−0.30	0.27
Unknown	0.002	0.13
Square of HGB*	−0.004	0.001
Nature Logarithm of ALP*	0.35	0.17
Inverse cube of INR*	−0.47	0.20

Abbreviations: ER, estrogen receptor; PR, progesterone receptor; HGB, hemoglobin; ALP, alkaline phosphatase; INR, international normalized ratio; SE, standard error.

^*^In order to calculate the prognostic index for the testing set, the missing data of HGB, ALP, and INR in the testing set were imputed as the mean values from the training set (after normality transformation, 154.57 for HGB, 4.24 for ALP, and 0.93 for INR, respectively).

### Model validation

**Figure 2:**
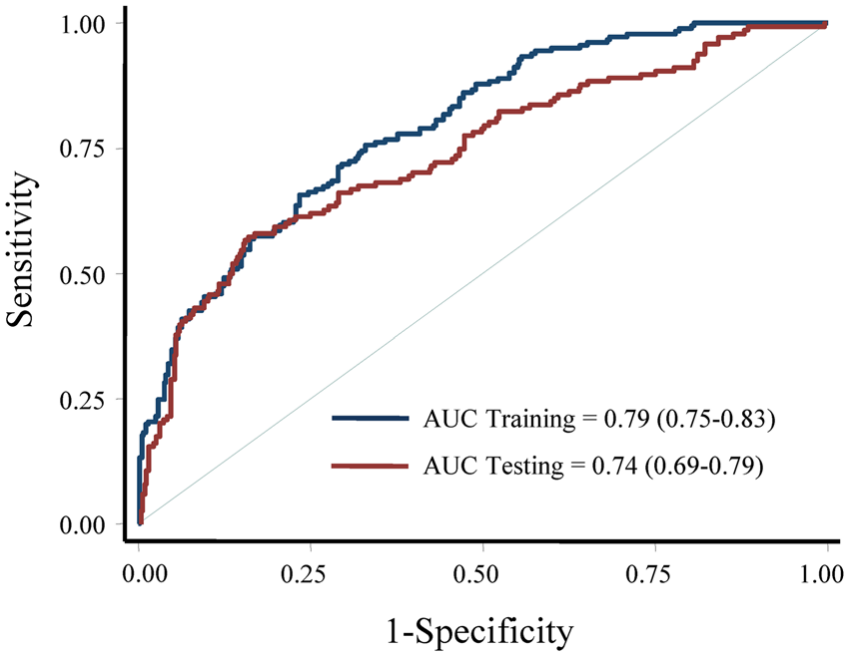
Assessment of model performance. The receiver operating characteristics curves were used to assess the performance of the final model in the training and testing sets.

The prognostic utility of the final model was measured by the area under the curve (AUC) of receiver operating characteristics (ROC) curve. The AUCs were 0.79 (95% CI: 0.75–0.83) and 0.74 (95% CI: 0.69–0.79) in the training and testing set, respectively (Fig. 2). We repeated the analyses after exclusion of the patients who were followed less than 3, 6, or 12 months. Increasing the length of the exclusion window minimizes potential confounding effects at the time of baseline sample collections. In the subset of patients who were followed ≥3 months, the AUCs in the training and testing sets were the same as that in the overall patients (Supplementary Figure S1A). Very similar results were observed in the subsets of patients who were followed either ≥6 or ≥12 months (Supplementary Figure S1B and C).

**Figure 3:**
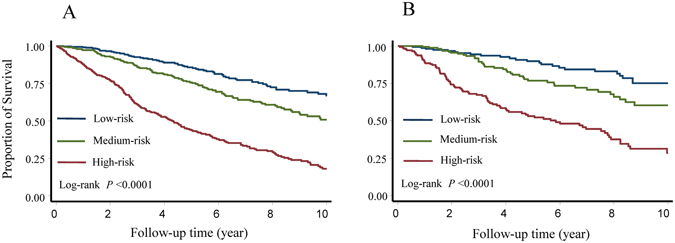
Disease free survival of different risk groups stratified by the final model. Kaplan-Meier survival estimates were used to characterize patients of different risk groups classified by the final model in the training (**A**) and testing (**B**) sets.

The patients were then classified into three risk groups according to the tertile distribution of the prognostic index. Compared with patients in the low-risk group, patients in the medium- and high-risk group had a significantly increased risk of recurrence with a hazard ratio (HR) of 1.75 (95% confidence interval [CI] 1.30–2.38) and 4.66 (95% CI 3.54–6.14), respectively in the training set (Table 4). The survivals were significantly different among these three risk groups (*P* < 0.0001, Fig. 3A). Similar results were found in the testing set (Table 4 and Fig. 3B), as well as, in the subset analyses (Supplementary Figure S2).

**Table 4:** Summary of disease free survival by risk category.

Groups	No. of patients disease free/recurrence	Median DFS time (year)	HR (95% CI)	Cox *P*	Log-rank *P*
**Training set**					
Low-risk	287/68	NR	1.00		<0.0001
Medium-risk	247/108	10.00 (8.14–11.93)	1.75 (1.30–2.38)	0.0003	
High-risk	149/205	4.26 (3.32–4.92)	4.66 (3.54–6.14)	<0.0001	
**Testing set**					
Low-risk	151/26	NR	1.00		<0.0001
Medium-risk	133/45	10.77 (8.77–12.51)	1.98 (1.22–3.21)	0.006	
High-risk	86/91	5.66 (3.72–7.43)	5.33 (3.44–8.27)	<0.0001	

Abbreviations and definitions: DFS, disease free survival; HR, hazard ratio; CI, confidence interval; NR, not reached.

## Discussion

In this study, we assessed the associations of a large panel of 33 laboratory variables available in routine clinical practice, with the DFS of a cohort of patients with breast cancer. Three laboratory variables were demonstrated to be associated with DFS and were used to construct a prognostic model that could be used to identify patients at risk of recurrence.

There is not widely accepted prognostic model based on objective criteria other than predicting survival using clinical features. In addition to demographic and basic clinical information, an increasing number of novel prognostic markers have been explored and identified^10–12^. However, the main problem for most of these studies is that biomarkers rely on sophisticated molecular and/or genetic tests^11, 13–16^. The practical application of the novel tests is inevitably restricted by its cost and complexity. Comparatively, the prognostic model developed in this study uses laboratory test results which have already been available as a consequence of routine clinical monitoring, at no incremental cost. Combining demographic and basic clinical information, together with these laboratory parameters, we developed a new prognostic model that may help physicians and patients estimate DFS and thereby inform medical decision-making and patient counseling.

Accumulating evidence has shown that black women have a high risk of breast cancer recurrence regardless of age and tumor size^17, 18^. We previously reported a racial disparity in breast cancer survival using the Jefferson Cancer Registry data^19^. In the current study, the risk of recurrence increased by 54% in African Americans compared to Caucasians, again demonstrating the prognostic value of race. Therefore, the inclusion of race, as well as other well-known predictors such as age, tumor characteristics, and treatments^20^ in the model makes the final model reliable in recurrence prediction and applicable in clinics. Our previous study also found that differences in tumor presentation and certain hematologic traits, for example HGB level were associated with racial disparity in breast cancer survival^19^. Abnormal metabolic index at baseline were reported to affect survival for all stages of breast cancer as well^21^. In the present study, three laboratory variables (HGB, ALP, and INR) which were significantly associated with patient DFS in univariate analyses were stepwise selected into the final model to predict patient survival. There are plausible physiological reasons why each of these variables might be an important predictor.

It is not uncommon for a cancer patient to have anemia. Besides radiotherapy and chemotherapy, cancer itself could cause anemia of chronic disease. The mechanism of anemia may be because of decreased lifetime of RBC, decreased sensitivity of bone marrow to erythropoietin, and decreased production of erythropoietin^22^. Not mentioning neoplasm itself has a higher need for nutrition, and some cytokines secreted by neoplasm cells could depress one’s appetite^23^, which may take parts in the development of cachexia, and devastating prognosis thereafter. A proportion of 62% to 71% breast cancer patients would have anemia during their courses of disease^24, 25^. The scale of anemia may accord to the phase of breast cancer and the medication of chemotherapy^26, 27^. Anemia, or HGB level, has been found to have strong relationship with recurrence and prognosis of breast cancer by the studies of ours and others^28–34^.

Bone is a common site of metastatic breast cancer. Skeletal isoenzyme of ALP increases when there is bone reconstruction. The mRNA of ALP expression elevates in cancer cells, and may participate in mammary mineralization just like ossification formed by osteoblast cells^35^. ALP is also a sensitive indicator of biliary blockage, and it is more reliable than other liver enzymes when there is a liver metastasis involved^36^. Therefore, it is reasonable that ALP, as a valuable prognostic marker, was selected in our final prognostic model. However, a recent study by Liu *et al*. failed to identify the association of pretreatment ALP level with overall survival in female Caucasian patients with non-metastatic invasive breast cancer^37^. It was reported previously that ALP may not increase much in early stage breast cancer patients, but there would be a significant increase in patients with metastatic disease^38^. Thus, the different findings between ours and Liu’s study may be due to the differences in patient characteristics (age, gender, and ethnicity) and cancer biology (cancer stage, histological types, and so on).

Tissue factor is a major participant of abnormal coagulation in cancer patients. The expression of tissue factor increases in many different neoplasm models, and has very strong relationship with severity and prognosis^39, 40^. Several studies have established connection between tissue factor and neoplasm growth and invasion^41–43^. Although breast cancer cells were reported to produce lower level of tissue factor compared to other cancer cell types^44^, high level of tissue factor was observed in studies focus more on chemotherapy of breast cancer patients when thrombosis was involved^45–47^. Tissue factor is not measured routinely, but factor VII function is often measured through PT or INR^48, 49^. So it may not be surprising that our final model including INR could be used to predict patient recurrence.

Several clinical tools have been developed to predict prognosis and survival benefit from treatments, using clinicopathological features, genetic profiles, and novel biomarkers^50^. In 466 invasive ductal carcinoma breast cancer patients, Volinia and Croce reported an AUC of 0.74 by integrating mRNA, microRNA, and DNA methylation next-generation sequencing data into the model^51^. Based on large database of microarray datasets, Griffith *et al*. developed a robust multi-gene mRNA transcription-based predictor of relapse free survival at 10 years, which achieved an AUC of 0.70 for hormone-positive node-negative breast cancer patients^52^. Using clinicopathological features and all 14 biomolecular signatures, Campbell *et al*. reported an AUC of 0.75 in early breast cancer patients, aiming to predict relapse-free survival^53^. Inevitably, molecular markers included in these studies added additional costs and limited clinical generalization. And apparently, those derived biomarkers which are not clinically certified, may exhibit large variations when measured in different laboratories. In comparison, the laboratory variables we used are inexpensive, readily available, and technically simple. Another prognostic index, the Nottingham Prognostic Index (NPI) is also widely used for predicting survival of operable primary breast cancer^54, 55^. NPI based on tumor size, histologic grade, and lymph node status^56^, although is simple and easily available in routine clinics, provided suboptimal performance in predicting patient recurrence^57–60^. The AUCs for NPI in our study were 0.66 and 0.63 in the training and testing sets, respectively (data not shown). Our current model including demographic and basic clinical variables, as well as 3 routine laboratory variables exhibited a prognostic power superior to previously reported models either using routine clinical variables, or using more expensive and complicated molecular biomarkers.

There are several strengths of our study. We had a relatively large population with 1,596 breast cancer patients and the final results were consistent between training and testing sets. We analyzed DFS of breast cancer patients after surgery to enhance the application of our model, given patients are at high risk of recurrence during the first 5 years of treatments. Generally, the measurements of laboratory variables around time of diagnosis are more relevant to a prediction model, however, are affected by factors such as treatments. Therefore, we restricted the analyses on laboratory variables measured within 3 months after surgery to minimize the influence of certain causes on the variables, such as less reliable test results due to longer time after surgery, or inaccurate test results due to adverse effects after treatments applied. Furthermore, compared to published survival models based on more specialized and expensive biomarkers identified by gene/protein expression assays, our current model relying on easily obtained hematological index from routine clinical practice exhibits a comparative prognostic performance but without increased cost. There are several limitations of this study. First, although our findings are internally validated and the selected variables in the final model are physiologically plausible, our cohort was from a single institution. The results from our current study should be further validated in large independent populations. Second, we collected the hematological indexes detected within 3 months after surgery and related records available in our medical charts. Some indexes which were examined during follow-ups at a long or irregular interval exhibited high percentages of missing values, possibly because they may only be requested to be tested when a clinical sign or side effect was detected or before a treatment-decision was made. Given the fact that request for tests may indirectly carry prognostic value, the missing information may possibly bias our finding. Although the multiple imputation method was used to estimate the missing values, it could neither provide an unbiased estimation nor eliminate potential confounding. Therefore, future studies are required to examine the model performance based on laboratory variables with more complete data. Third, because we do not know whether the patients who were censored due to loss to follow-up were as likely to have a subsequent event as those individuals who remained in the study, informative censoring may occur and bias the results^61, 62^. Fourth, some important factors such as HER2 status and target therapy were not included in the final model due in part to the missing data. Considering that HER2 is also essential for making treatment decision, and target therapy in HER2 positive patients could affect patient survival, further study could explore the performance of a model incorporating these two variables. Fifth, the patients included in the study were diagnosed between 1988 and 2011. Changes of diagnosis criteria and treatment regimens in this relatively long time period might increase the heterogeneity of our population. Sixth, we excluded the patients due to the lack of laboratory variable measurement, which might confound the results. However, when we compared the basic demographic and clinical characteristics between the included and excluded patients, we did not find significant difference in most of these variables (data not shown), indicating that the confounding, if there is any, may be minor. Moreover, we excluded some patients according to a given clinical characteristics, for example, without surgery. This study design, although made the study population more homogeneous, might restrict the generalization of our final model. Finally, this model performs well as a prognostic model to predict DFS of all patients once identified as breast cancer, but there is a lack of efficiency on predicting the responses to treatments that were used afterwards. This prediction model can be better developed if the follow-up and evaluation of treatments at different time point are included in the analyses.

In summary, we developed an inexpensive model that was mainly based on readily available objective data for a cohort of breast cancer patients identified and treated in a single-institute. If further validated, this model could be used to identify breast cancer patients who are at high risk of recurrence and be helpful to motivate individuals to pursue benefits from treatments.

## Methods

### Study population

Based on the electronic medical records from the Cancer Registry at Thomas Jefferson University Hospital, we identified histologically confirmed female breast cancer patients who were diagnosed and/or treated from October, 1988 to December, 2011^19^. For the analyses in this study, we excluded the patients (i) without mastectomy or breast conservation surgery and/or without routine blood tests within 3 months after surgery; (ii) with 0/unknown stage and/or cancer histology of carcinoma *in situ* (including ductal carcinoma *in situ* and lobular carcinoma *in situ*); (iii) without recurrence information or never disease free. Finally, a cohort of 1,596 breast cancer patients was selected based on these criteria (Fig. 1). This study was approved by the Institutional Review Board (IRB) at the Thomas Jefferson University. Because this study was based on data obtained from the review of archived medical charts, patient consent was waived by the IRB of the Office of Human Research in Thomas Jefferson University under an approved protocol including the approval for the request for waiver of authorization to collect protected health information.

### Data collection

Demographic variables including age, race/ethnicity, smoking status, and drinking status were collected in this study. Basic clinical variables included tumor size, stage, grade, histology, lymph nodes metastatic rate, ER status, PR status, and treatments (hormone therapy, chemotherapy, and radiation therapy). Routine blood-based laboratory test data were also obtained from medical charts, which included a total of 33 variables in four categories: complete blood count (CBC), comprehensive metabolic panel (CMP), coagulation panel, and leukocyte differentiation tests (Supplementary Table S1). Following 10 variables were included in the CBC panel: white blood cell (WBC), red blood cell (RBC), hemoglobin (HGB), hematocrit (HCT), mean corpuscular volume (MCV), mean corpuscular hemoglobin (MCH), mean corpuscular hemoglobin concentration (MCHC), red cell distribution width (RDW), mean platelet volume (MPV), and platelet count. Routine CMP panel recorded 10 variables including blood urine nitrogen (BUN), creatinine, glucose, protein, albumin, alkaline phosphatase (ALP), alanine aminotransferase (ALT), aspartate aminotransferase (AST), total bilirubin, and anion gap. The coagulation condition of each patient was evaluated by prothrombin time (PT), partial thromboplastin time (PTT), and international normalized ratio (INR). The percentages of neutrophils, lymphocytes, monocytes, basophils, eosinophils as well as their absolute numbers were all obtained from the results of a test for leukocyte differentiation.

### Statistical analysis

#### General analytic strategy

SAS (Version 9.2, SAS institute, Cary, NC) and Stata (Version 12.0, Stata Corp., College station, TX) software packages were used for data analyses in this study. The clinical endpoint analyzed in this study was DFS. The definition of recurrence was that after surgical removal of primary tumor, the regrowth of tumor in the original site or regional lymph nodes, or distant organs. DFS was defined as the time from surgery to the first event of either recurrence or death^63^. Patients who were alive and recurrence-free on December 31, 2011 were censored. Patients who were lost to follow-up were censored as well. In routine blood-based laboratory tests, variables with greater than 50% missing observations were excluded from analyses. For patients with multiple measurements of the same variable, the mean value of these measurements were calculated and used in the analyses. To develop a risk prediction model, patients were sorted by surgery date and two of every three sorted patients were included in the training set. The remaining patients were included in the testing set to internally assess the predictive performance and control overfitting of the model^64, 65^. All statistical tests were two-sided, and a *P*-value of less than 0.05 was considered statistically significant.

#### Identification of candidate variables

Comparisons of demographic, clinical, and laboratory variables between training and testing sets were performed using the *chi*-square test for categorical variables and Student’s *t* test for continuous variables. The association between each variable and patient DFS was assessed using Kaplan-Meier and Cox proportional hazards regression analyses in the training set. Variables that demonstrated a significant association with DFS were included in the next stepwise selection and model construction. Laboratory variables had to be significant in all of the categorical, continuous, and log-rank analyses. Bootstrap resampling method is used to internally validate the analyses of these results. A total of 1,000 bootstrap samples were generated for each analysis. Each time a bootstrap was drawn from the original dataset and the *P*-value for the analysis was calculated. The number of times with a *P*-value less than 0.05 was counted.

#### Stepwise selection and model construction

In order to minimize the confounding effects resulting from potential high correlations between laboratory variables and demographic and clinical variables, we forced significant demographic and clinical variables from the univariate analysis into the model. For the laboratory variables, we conducted stepwise selection using multivariate Cox proportional hazards model with significant laboratory variables identified in the univariate analysis. All continuous variables were kept continuous in the multivariate Cox regression and model construction process to avoid loss of power and residual confounding^66^. Multiple imputation method was used to handle the missing data in the training set^67^. The 10 imputation datasets from the training set were generated by Stata’s MI package, basing on the multivariate normal imputation^68^. And the missing data in the testing set were imputed as the mean values from the training set. Before imputation, box-cox method was used to transform variables with skewed distribution toward normality. In each imputed dataset, a forward stepwise selection was conducted using Akaike’s information criterion (AIC) which balances the data fitting and complexity of the model and reduced risk of overfitting^69^. The model with the smallest AIC was selected as the best model for each imputed dataset. The significant demographic and clinical variables were forced into the final model which was derived from each of the 10 imputed dataset as a composite. A laboratory variable which was selected in at least 6 imputed datasets was included in the final model. The parameter estimate (weight/coefficients) of each variable was calculated based on the pooled imputed datasets. A prognostic index was derived by calculating the sum of each variable multiplied by its corresponding weight in the final model.

#### Model validation

Two methods were used for model validation and applied in both training and testing sets. Model’s capability to predict recurrence was assessed by constructing the ROC curves and calculating the AUCs^70^.

In the second validation method, patients were classified into three risk groups based on the prognostic index calculated by the model. The cutoff value was determined by tertile distribution of the prognostic index. HRs with 95% CI in different risk groups were assessed by Cox proportional hazards model. Survival curves were plotted using Kaplan-Meier method and compared using the log-rank test.

## Electronic supplementary material

Supplementary Information
