# Supplementary material for: Prognostic value of routine laboratory variables in prediction of breast cancer recurrence
Source: Sci Rep. 2017 Aug 15;7:8135. doi: 10.1038/s41598-017-08240-2 (PMC5557903; doi:10.1038/s41598-017-08240-2)
Supplement: Supplementary file 1 — Supplementary Information [file 41598_2017_8240_MOESM1_ESM.pdf]

Prognostic value of routine laboratory variables in prediction of breast cancer recurrence

Zhu Zhu[1,2], Ling Li[2], Zhong Ye[2], Tong Fu[1], Ye Du[1], Aiping Shi[1], Di Wu[1], Ke Li[3], Yifan Zhu[2], Chun Wang[2,4,*] & Zhimin Fan[1,*]

Supplementary Table S1. Values of laboratory variables in the training and testing sets.

Panels	Training set				Testing set			***P***
**Variables (unit)**	**N**	**Mean**	**SD**		**N**	**Mean**	**SD**	**value**
**Complete Blood Count**								
**HCT** (%)	1026	37.05	4.33		520	37.08	4.21	0.90
**HGB** (g/dL)	1027	12.34	1.50		520	12.31	1.43	0.71
**MCH** (pg)	1031	29.89	2.35		523	29.76	2.25	0.28
**MCHC** (g/dL)	1026	33.31	1.11		520	33.21	1.14	0.11
**MCV** (fL)	1027	89.68	5.88		520	89.53	5.68	0.64
**Platelet** (B/L)	1027	260.84	67.72		520	259.54	64.75	0.72
**MPV** (fL)	608	10.72	0.91		304	10.75	0.89	0.72
**RBC** (T/L)	1032	4.15	0.56		520	4.16	0.49	0.88
**RDW** (%)	1026	13.81	1.60		520	13.87	1.65	0.53
**WBC** (B/L)	1028	7.48	2.59		520	7.66	3.29	0.28
**Comprehensive Metabolic Panel**								
**Albumin** (g/dL)	680	4.28	0.39		339	4.28	0.39	0.79
Protein(g/dl)	301	6.28	1.71		158	6.39	1.61	0.49
**ALP** (IU/L)	738	74.86	43.17		374	75.09	29.31	0.92
**ALT** (IU/L)	616	23.91	18.33		326	22.34	10.44	0.91
**AST** (IU/L)	739	25.42	13.62		374	24.20	8.13	0.75
**Total Bilirubin** (mg/dL)	722	0.60	0.26		372	0.57	0.21	0.15
**Anion Gap** (mmol/L)	955	8.51	2.05		480	8.39	1.97	0.30
**Creatinine** (mg/dL)	963	0.84	0.45		485	0.84	0.34	0.99
Glucose (mg/dL)	400	105.71	40.97		207	111.93	46.86	0.11
**Urea Nitrogen** (mg/dL)	957	14.72	6.04		481	14.90	6.53	0.62
**Coagulation Panel**								
**INR**	640	1.06	0.19		315	1.05	0.18	0.42
**PT**	577	14.30	5.18		282	13.88	2.02	0.09
**PTT**	631	30.64	6.17		315	30.1	6.61	0.94
**Leukocyte Differentiation Test**								
Neutrophils	428	65.87	12.02		203	65.94	11.67	0.94
Lymphocytes	426	23.24	10.21		203	23.43	9.59	0.83
Monocytes	426	8.61	3.63		203	8.20	3.68	0.19
Eosinophils	426	1.86	1.65		203	1.92	1.93	0.65
Basophils	426	0.49	0.41		203	0.48	0.45	0.91
Absolute Neutrophils	393	5.47	2.79		185	5.51	2.58	0.87
Absolute Lymphocytes	393	1.60	0.66		185	1.64	0.69	0.48
Absolute Monocytes	393	0.60	0.25		185	0.58	0.29	0.42
Absolute Eosinophils	393	0.13	0.13		185	0.13	0.14	0.79
Absolute Basophils	393	0.02	0.03		185	0.02	0.03	0.69

Abbreviations: HCT, hematocrit; HGB, hemoglobin; MCH, mean corpuscular hemoglobin; MCHC, mean corpuscular hemoglobin concentration; MCV, mean corpuscular volume; MPV, mean platelet volume; RBC, red blood cell; RDW, red cell distribution width; WBC, white blood cell; ALP, alkaline phosphatase; ALT, alanine amino transferase; AST, aspartate amino transferase; INR, international normalized ratio; PT, prothrombin time; PTT, partial thromboplastin time; SD, standard deviation. Variables which are valid for further analyses are shown in bold.

Supplementary Table S2. Univariate analysis of the associations of demographic and basic clinical variables with patient disease-free survival in the training set.

**Variables**	**Level**	**No. of patients disease free /recurrence**	***P***	**HR (95% CI)**	**Cox *P***	**Log-rank *P***
Age	<58	361/156	0.0002	1.00		<0.0001
	≥58	322/225		1.53 (1.25-1.88)	<0.0001	
Race	Caucasian	512/265	0.03	1.00		0.001
	African American	121/93		1.54 (1.22-1.96)	0.0003	
	Others	50/23		1.05 (0.68-1.60)	0.84	
Smoking status	Never	354/217	0.09	1.00		0.94
	Current	77/47		0.97 (0.71-1.33)	0.86	
	Former	140/75		0.96 (0.74-1.24)	0.74	
	Unknown	112/42		0.90 (0.65-1.26)	0.55	
Drinking status	Never	317/165	0.02	1.00		0.52
	Ever	228/158		0.89 (0.71-1.10)	0.28	
	Unknown	138/58		0.88 (0.66-1.21)	0.46	
Tumor stage	Stage I	412/154	<0.0001	1.00		<0.0001
	Stage II	227/128		1.38 (1.09-1.74)	0.008	
	Stage III	40/72		3.29 (2.49-4.36)	<0.0001	
	Stage IV	4/27		8.28 (5.49-12.50)	<0.0001	
Tumor grade	Well	90/31	<0.0001	1.0		0.0003
	Moderately	290/120		0.89 (0.60-1.32)	0.55	
	Poorly	219/176		1.46 (0.99-2.13)	0.05	
	Not determined	84/54		1.22 (0.79-1.90)	0.37	
Tumor histology	Invasive ductal carcinoma	557/311	0.31	1.00		0.15
	Invasive lobular carcinoma	44/30		1.18 (0.82-1.73)	0.37	
	Mixed carcinoma	74/32		0.81 (0.56-1.16)	0.24	
	Other	8/8		1.82 (0.91-3.69)	0.09	
Tumor size	No mass/unknown	171/75	<0.0001	1.00		<0.0001
	2-9mm	129/52		0.63 (0.44-0.90)	0.01	
	10-29mm	294/153		0.72 (0.54-0.95)	0.02	
	30-49mm	61/45		1.04 (0.72-1.50)	0.84	
	50-99mm	24/47		2.17 (1.50-3.12)	<0.0001	
	≥10cm	4/9		2.39 (1.20-4.76)	0.01	
Lymph nodes metastatic rate	0%	420/171	<0.0001	1.00		<0.0001
	1-20%	79/49		1.39 (1.01-1.91)	0.04	
	20-49%	34/21		1.33 (0.84-2.09)	0.22	

	50-79%	18/14		1.95 (1.13-3.37)	0.02	
	80-100%	12/22		3.71 (2.37-5.79)	<0.0001	
	Not determined	102/74		2.23 (1.70-2.93)	<0.0001	
	Unknown	18/30		2.03 (1.38-2.99)	0.0003	
ER status	Negative	131/92	<0.0001	1.00		0.008
	Positive	500/234		0.69 (0.54-0.88)	0.003	
	Unknown	52/55		0.87 (0.62-1.22)	0.42	
PR status	Negative	184/129	<0.0001	1.00		0.0007
	Positive	446/196		0.66 (0.53-0.82)	0.0002	
	Unknown	53/56		0.86 (0.63-1.18)	0.36	
Chemotherapy	No	399/219	0.20	1.00		0.05
	Yes	259/155		1.29 (1.05-1.59)	0.02	
	Unknown	25/7		0.96 (0.45-2.04)	0.91	
Radiation therapy	No	364/239	0.01	1.0		0.01
	Yes	294/133		0.73 (0.59-0.91)	0.004	
	Unknown	25/9		1.26 (0.65-2.45)	0.50	
Hormone therapy	No	388/264	0.0002	1.0		0.01
	Yes	240/99		0.79 (0.63-1.00)	0.045	
	Unknown	55/18		0.55 (0.34-0.89)	0.015	

Abbreviations: ER, estrogen receptor; PR, progesterone receptor; HR, hazard ratio; CI, confidence interval.

Supplementary Table S3. Number of times variable was selected out of 10 imputation datasets.

Variables	Number of times variable was selected out of 10 imputation datasets	Transfer[1]
HCT	1	2
HGB	9	2
RBC	0	-0.5
RDW	0	-3
Albumin	0	3
ALP	6	0
INR	7	-3
PT	3	-3

[1]The following transformations were made for continuous variables in the model: 3 = cube, 2 = square, 0 = natural log, -3 = inverse cube, -0.5 = inverse square root.

Abbreviations: HCT, hematocrit; HGB, hemoglobin; RBC, red blood cell; RDW, red cell distribution width; ALP, alkaline phosphatase; INR, international normalized ratio; PT, prothrombin time.

Supplementary Table S4. Estimation of prognostic index based on the model developed in this study.

Variables	Enter Value
Age	80
Race	African American
Stage	2
Tumor size (mm)	8
Lymph nodes metastatic rate	No nodes examined
ER status	Negative
PR status	Negative
Chemotherapy	Yes
Radiation therapy	No
Hormone therapy	No
Transferred HGB	153.76
Transferred ALP	4.26
Transferred INR	0.68
Prognostic index	3.54

Low risk group: prognostic index ≤ 1.36; Median risk group: prognostic index 1.36-1.99; High risk group: prognostic index > 1.99

Abbreviations: ER, estrogen receptor; PR, progesterone receptor; HGB, hemoglobin; ALP, alkaline phosphatase; INR, international normalized ratio.

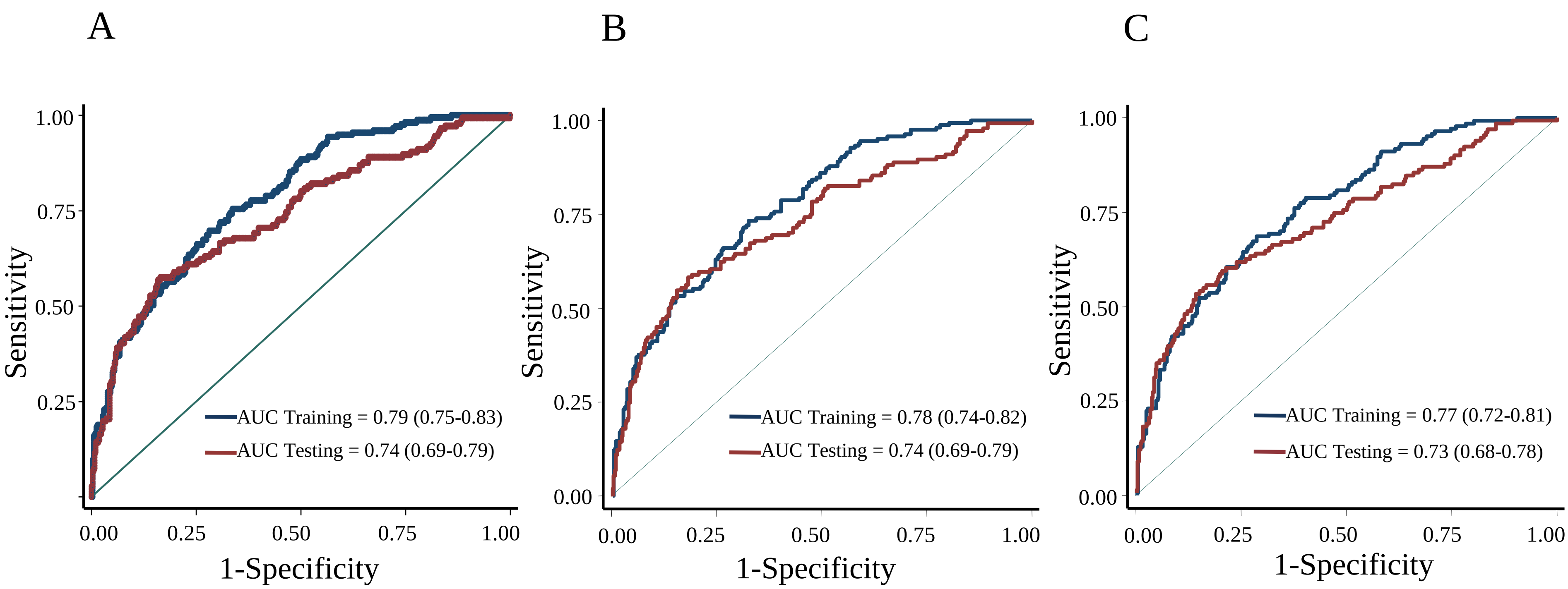

Supplementary Figure S1. Assessment of model performance in subsets of patients. The patients were followed ≥ 3 months (A), ≥ 6 months (B), or ≥ 12 months (C).

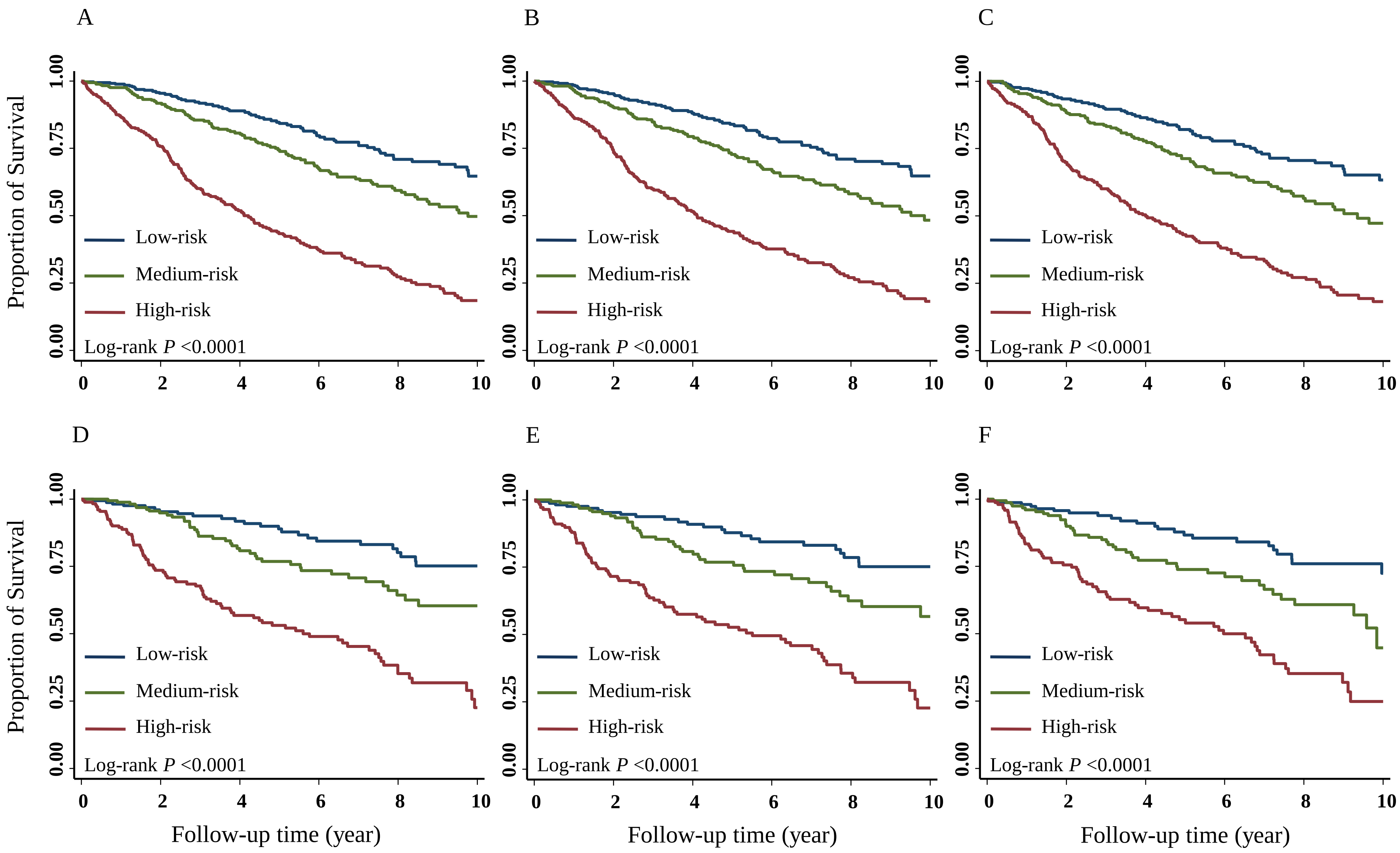

Supplementary Figure S2. Disease free survival of different risk groups stratified by the final model in subsets of patients. The survival curves were compared among different risk groups in the subsets of patients who were followed ≥ 3 months (A), ≥ 6 months (B), or ≥ 12 months (C) in the training set, and in the subsets of patients who were followed ≥ 3 months (D), ≥ 6 months (E), or ≥ 12 months (F) in the testing set, respectively. The time point zeros indicate the time starting from 3 months (A and D), 6 months (B and E), or 12 months (C and F) after surgery.
